# Effects of the Small Molecule HERG Activator NS1643 on Kv11.3 Channels

**DOI:** 10.1371/journal.pone.0050886

**Published:** 2012-11-30

**Authors:** Arne Bilet, Christiane K. Bauer

**Affiliations:** Institute of Cellular and Integrative Physiology, University Medical Center Hamburg-Eppendorf (UKE), Hamburg, Germany; University of Texas Health Science Center, United States of America

## Abstract

NS1643 is one of the small molecule HERG (Kv11.1) channel activators and has also been found to increase erg2 (Kv11.2) currents. We now investigated whether NS1643 is also able to act as an activator of Kv11.3 (erg3) channels expressed in CHO cells. Activation of rat Kv11.3 current occurred in a dose-dependent manner and maximal current increasing effects were obtained with 10 µM NS1643. At this concentration, steady-state outward current increased by about 80% and the current increase was associated with a significant shift in the voltage dependence of activation to more negative potentials by about 15 mV. In addition, activation kinetics were accelerated, whereas deactivation was slowed. There was no significant effect on the kinetics of inactivation and recovery from inactivation. The strong current-activating agonistic effect of NS1643 did not result from a shift in the voltage dependence of Kv11.3 channel inactivation and was independent from external Na^+^ or Ca^2+^. At the higher concentration of 20 µM, NS1643 induced clearly less current increase. The left shift in the voltage dependence of activation reversed and the voltage sensitivity of activation dramatically decreased along with a slowing of Kv11.3 channel activation. These data show that, in comparison to other Kv11 family members, NS1643 exerts distinct effects on Kv11.3 channels with especially pronounced partial antagonistic effects at higher concentration.

## Introduction

The ether-a-go-go-related gene (erg) or Kv11 K^+^ channel family consists of three family members which differ in their voltage dependence and gating kinetics, but also share unique properties like a peculiar gating behaviour, an unusual sensitivity to external K^+^ or a high affinity block by class III antiarrhythmic drugs [1], [2], for review see [3]. In contrast to the widespread distribution of Kv11.1 channels in many excitable tissues, Kv11.2 and Kv11.3 channels are mainly expressed in the central nervous system and therefore, they have often been referred to as “neuronal” Kv11 channels [1]. The three Kv11 channels exhibit a distinct, but partially overlapping distribution in the brain, with Kv11.1 and Kv11.3 being considerably more abundant than Kv11.2 [4], [5], [6]. All three Kv11 channel subunits may be present in a single cell, as shown for mitral cells in the olfactory bulb [7], enabling the formation of heteromultimeric Kv11 channels [8]. Other brain regions exhibit a dominant expression of one Kv11 subunit, like Kv11.1 in brain stem nuclei or Kv11.3 in hippocampal pyramidal cells [4].

The electrophysiological characterization of functional Kv11 channels outside the heart is growing slowly and covers studies on neuronal, endocrine or smooth muscle cells (reviewed in [9]). In these tissues, Kv11 channels can modulate excitability by contributing to the maintenance of the resting membrane potential or to the phenomenon of action potential frequency accommodation (e.g. [10], [11], [12], reviewed in [3], [9]). Especially the Kv11.3 channel with a more negative activation threshold and faster activation kinetics than Kv11.1 [1] is well suited to contribute to both, resting potential and spike frequency adaptation.

Due to their expression in the heart and their functional importance for the repolarization of the human cardiac action potential, Kv11.1 (or HERG, human erg) channels have been investigated in detail concerning their biophysical and pharmacological properties. In the human heart, reduced function of the repolarizing Kv11.1 current I_Kr_ either by genetic mutations or more commonly by pharmacological block results in a prolongation of the ventricular action potential possibly inducing a long QT syndrome type 2 (LQT2) which increases the risk for life-threatening torsade de pointes arrhythmia [9].

In the last few years several “HERG activators” were developed which increase the Kv11.1 current by different alterations of the biophysical properties of the channels (reviewed in [13]). The small molecule compound NS1643 has been shown to activate native and heterologously expressed Kv11.1 channels [14], [15], [16], [17], [18], [19] as well as Kv11.2 channels [20]. So far, the effects of NS1643 on Kv11.3 channels had not been investigated, but another small molecule HERG activator, the compound RPR26024, exhibited a striking lack of activating effects on Kv11.3 [21], [22].

We have now analyzed the actions of NS1643 on Kv11.3 channels heterologously expressed in CHO cells and found that the drug is also able to activate Kv11.3 channels by qualitatively similar changes in the biophysical properties as described for Kv11.1 channels in mammalian expression systems [18]. In contrast to its agonistic activity, the partial antagonistic actions of NS1643 on Kv11.3 channels were especially pronounced and accompanied by unique changes in the voltage sensitivity of the channel.

## Materials and Methods

### Heterologous Expression

Chinese hamster ovary (CHO) cells were cultured in DMEM (Gibco/Invitrogen GmbH, Karlsruhe, Germany) supplemented with 1% penicillin-streptomycin-glutamine (Gibco) and 10% fetal calf serum (Biother, Kelkheim, Germany) at 37°C in a humidified incubator (95% air, 5% CO_2_). For heterologous Kv11.3 channel expression, CHO cells were plated onto poly-D-lysine-coated coverslips and transfected with rat Kv11.3 cDNA (1 ng/µl) and cDNA encoding EGFP-N1 (0.5–0.8 ng/µl; Clontech, Heidelberg, Germany) using LipofectAMINE 2000 reagent (Invitrogen, Karlsruhe, Germany) according to manufacturer`s instructions.

### Electrophysiology

Experiments were performed 8–48 hours after CHO cell transfection in the conventional whole-cell configuration of the patch-clamp technique. Patch pipettes had resistances of 2 to 4 MΩ. Data were low-pass filtered at 3 kHz and compensated for both fast and slow capacity transients. The access resistance ranged from 2.5 to 10 MΩ and series resistance was compensated for by 60 to 90%. Data were not corrected for the liquid junction potential error (∼4 mV with external Ringer solution). An EPC-9 patch clamp amplifier was used in combination with the PULSE stimulation and data acquisition software (HEKA, Lamprecht, Germany). All experiments were performed at room temperature.

### Solutions and Chemicals

The external Ringer solution contained (in mM): NaCl 140, KCl 5, MgCl_2_ 0.8, CaCl_2_ 1, HEPES 10, glucose 5; pH adjusted to 7.35 with NaOH. In one set of experiments using external “low Ca^2+^ solution”, the free Ca^2+^ concentration was reduced to about 70 nM by adding 2.5 mM EGTA to the Ringer solution. In the external “0 Na^+^ solution”, the NaCl of the Ringer solution was completely replaced by NMDG with Cl^-^ as anion derived from pH adjustment with HCl. The standard pipette solution contained (in mM): KCl 140, MgCl_2_ 2, CaCl_2_ 1, HEPES 10, EGTA 2.5; pH was adjusted to 7.3 with KOH. NS1643 (1,3-bis-(2-hydroxy-5-trifluoromethyl-phenyl)-urea) was from NeuroSearch A/S (Ballerup, Denmark) and purchased from Biozol (Eching, Germany). Prior to the experiments, stock solutions of 30 mM NS1643 (in DMSO) were dissolved in external solution to yield the final drug concentrations ranging from 0.1 to 20 µM NS1643.

### Data Analysis

Experimental data are given as means ± SEM, with *n* representing the number of experiments from different cells. Student´s two-tailed paired or unpaired *t* test was used to test for significance. Data processing was performed with PulseFit 8.65 (HEKA, Lamprecht, Germany), Excel (Microsoft Corp., Redmont, WA), Sigmaplot 11.0 (Systat Software, Inc., San Jose, CA) and IGOR Pro 4.0 (Wavemetrics, Portland, OR).

To assess the voltage dependence of Kv11.3 channel activation, normalized data were fitted with a Boltzmann equation: 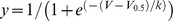, where *V_0.5_* is the potential of half-maximal current amplitude and *k* is the slope factor. The time course of activation was determined by an envelope-of-tail protocol. Tail current amplitudes were normalized to the maximal amplitude and, apart from the first two or three data points, data were fitted with a single exponential function yielding the time constant of activation. The time constant of inactivation and the time constant of recovery from inactivation were obtained by fitting single exponential functions to the recorded current traces. The time constant of fast deactivation (τ_deact_) was obtained in the voltage range between −90 and −120 mV by fitting the decaying part of the inwardly directed Kv11.3 current traces either with one or with the sum of two exponential functions.

## Results

### Dose-dependent Effects of NS1643 on Kv11.3 Channel Activation

**Figure 1:**
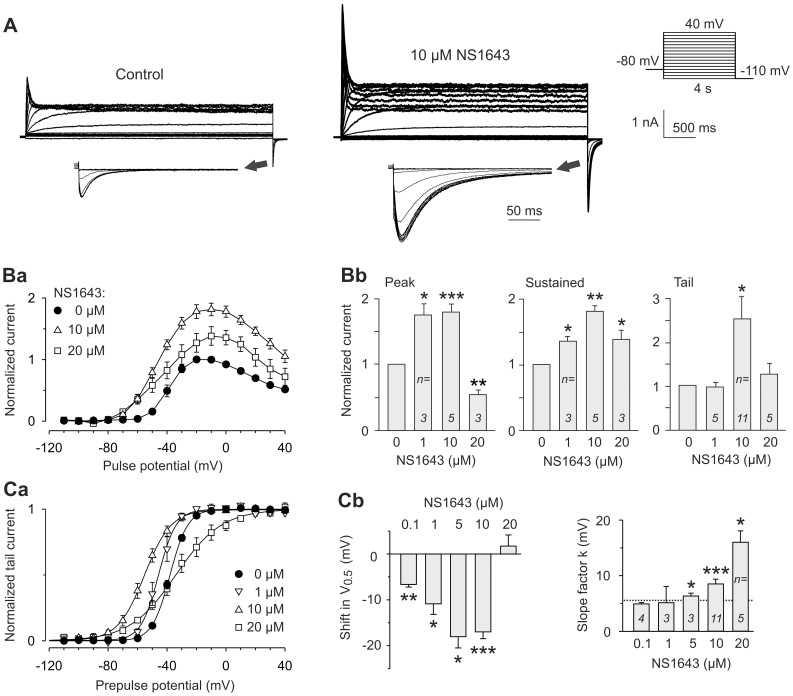
Dose-dependent effects of NS1643 on Kv11.3 current amplitude and voltage-dependent activation. Membrane currents were recorded in CHO cells transiently expressing Kv11.3 channels. Using a holding potential of −80 mV, channel activation was assessed by 4 s test pulses between −110 mV and +40 mV with 10 mV increments, followed by a constant pulse to −110 mV for 250 ms (see pulse diagram). (A) Representative Kv11.3 current traces measured in the same cell before (control) and after application of 10 µM NS1643. Tail currents are also shown with tenfold expanded time scale. (Ba) Current-voltage relationship of outward current amplitudes measured at the end of the depolarizing pulses under control conditions and after application of 10 µM (*n* = 5) or 20 µM NS1643 (*n* = 3). For each experiment, current amplitudes were normalized to the maximum of the respective control. (Bb) Effects of different concentrations of NS1643 on Kv11.3 peak current (at +40 mV), maximal steady-state current (at −10 or −20 mV) and maximal tail current amplitude. Current amplitudes were normalized to the respective control values. (Ca) Peak tail current amplitudes plotted against the potential of the preceding test pulse. Data for control experiments and measurements obtained in the presence of different concentrations of NS1643 were normalized to the maximum tail current amplitudes, averaged and fitted with a Boltzmann function, yielding the activation curves with the potentials of half-maximal activation, *V_0.5_*, and the slope factor *k*. (Cb) Mean NS1643-induced changes in *V_0.5_* and absolute slope factors *k* obtained from experiments with application of different concentrations of NS1643. The dashed line indicates the mean *k* value of the controls. Data are means ± SEM and asterisks indicate significance versus paired control data: *p<0.05, **p<0.01, ***p<0.001.

Kv11.3 currents were recorded from transiently transfected CHO cells before and after application of different concentrations of NS1643. Fig. 1A shows representative Kv11.3 membrane currents recorded under control conditions and in the presence of 10 µM NS1643. The figure demonstrates the enhanced Kv11.3 currents under the influence of 10 µM NS1643. The outward currents were measured at the end of the variable test pulse, normalized to the maximum current amplitude of the control and plotted against the test pulse potential (Fig. 1Ba). Under control conditions, outward currents were evoked at depolarizing test pulse potentials more positive than −60 mV. The amplitude of these Kv11.3 currents increased up to potentials between −20 and −10 mV. Typical for Kv11.3 channels, more positive test pulses induced increasing initial current transients followed by lower sustained currents, resulting in a bell-shaped current-voltage relationship of the Kv11.3 steady-state current. The most pronounced increase in Kv11.3 steady-state current was observed with application of 10 µM NS1643, whereas the higher concentration of 20 µM NS1643 induced a less intense current increase. Correspondingly, the maximal activating effect of NS1643 on peak and tail currents was found at a concentration of 10 µM (Fig. 1Bb). Nevertheless, the dose dependence of the drug effects on Kv11.3 differed between peak, tail and steady-state currents: 1 µM NS1643 already exerted a huge increase in peak current amplitude, but failed to significantly enhance tail currents. Application of the high concentration of 20 µM NS1643 did not result in a significant tail current increase and even suppressed the Kv11.3 peak current component.

The effects of NS1643 on the voltage dependence of Kv11.3 channel activation were determined by plotting the peak tail current amplitudes against the prepulse potential (Fig. 1Ca). Boltzmann functions were fitted to the data points to determine the potential of half-maximal Kv11.3 channel activation (V_0.5_) and the slope factor (*k*). Up to 10 µM, the NS1643-induced Kv11.3 current increase was accompanied by a significant shift in the voltage dependence of activation to more negative potentials. Prior to the application of NS1643, the control measurements yielded a V_0.5_ value of −37.9±0.8 mV and a slope factor *k* of 5.8±0.3 mV (*n* = 20). The left shift in half-maximal activation was concentration-dependent and ranged from about 7 mV at 0.1 µM NS1643 to more than 15 mV with 5 and 10 µM NS1643 (Fig. 1Cb). Application of a higher concentration, i.e. 20 µM NS1643, exerted rather different effects. While the potential of half maximal current activation was no longer shifted to the left, the slope factor *k* dramatically increased to 15.4±2.2 mV (*n* = 5) reflecting a strong reduction in the voltage sensitivity of the Kv11.3 channels. Even less pronounced, the decrease in the slope of the activation curves was already present with the smaller NS1643 concentrations of 5 and 10 µM (Fig. 1Cb). These data demonstrate that the net effect on Kv11.3 macroscopic current results from different changes in biophysical properties caused by NS1643.

### Effect of NS1643 on Kv11.3 Activation Kinetics

**Figure 2:**
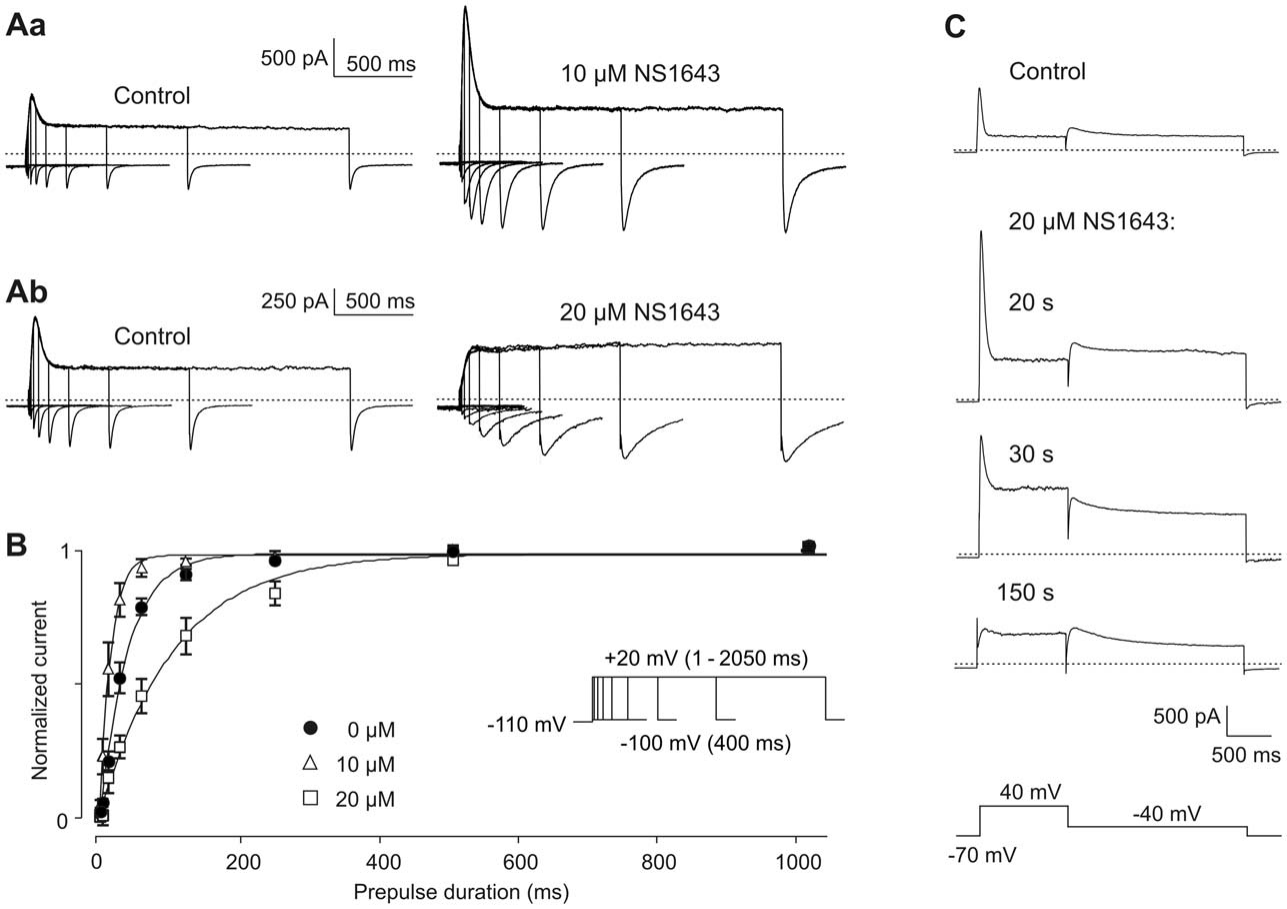
Effect of NS1643 on the time course of activation of Kv11.3 channels. Membrane currents were evoked by depolarizing pulses to +20 mV of increasing duration (between 1 and 2050 ms), followed by a 400 ms hyperpolarization to −100 mV. The holding potential was −80 mV and each pulse sequence was preceded by a 2 s hyperpolarization to −110 mV. (A) Representative membrane currents recorded before and after application of 10 µM (Aa) or 20 µM (Ab) NS1643. (B) Normalized peak tail current amplitudes were averaged, plotted against the duration of the preceding depolarizing pulse, and fitted with single exponential functions. Data are means ± SEM from paired experiments (*n* = 5 for 10 µM, *n* = 3 for 20 µM NS1643). (C) Time course of the effects of 20 µM NS1643. Kv11.3 currents were elicited before and various time after the start of NS1643 application from a holding potential of −70 mV. Each pulse sequence consisted of a 1 s pulse to 40 mV, followed by a 2-s step to −40 mV to elicit tail currents. 20 µM NS1643 was applied after control runs of the test pulse sequence, which was repeated every 10 s.

Kv11.3 activation kinetics was assessed by an envelope of tail protocol. Starting from a holding potential of −80 mV, the depolarizing pulses of variable duration to +20 mV was always preceded by a 2 s hyperpolarization to −110 mV in order to ensure complete Kv11.3 channel deactivation. Each pulse sequence ended with a hyperpolarization to −100 mV to elicit inward tail currents which mirrored the fraction of Kv11.3 channels activated during the course of the depolarizing pulse. Representative Kv11.3 current traces as well as averaged data before and after application of 10 µM or 20 µM NS1643 are illustrated in Fig. 2. At a concentration of 10 µM, NS1643 significantly increased the rate of Kv11.3 channel activation, whereas it slowed the rate of activation at a concentration of 20 µM (control: τ_activation_ = 47.5±4.6 ms, *n* = 8; 10 µM NS1643: τ_activation_ = 21.7±5.2 ms; *n* = 5, *p* = 0.004; 20 µM NS1643: τ_activation_ = 156.0±20.6 ms; *n* = 5, *p*<0.0001).

Fig. 2C illustrates that the time course of the effects of 20 µM NS1643 seemed to parallel the effects of increasing drug concentrations. 20 s after bath application, a clear current activation was evident with a strong increase in the peak as well as the sustained current amplitude. With longer presence of the drug, the antagonistic effects took over: the increase in sustained current partially reversed and the initial current peak almost disappeared. The appearance and magnitude of an initial Kv11 current transient depends on the relative rates of channel activation and inactivation [1]. Therefore, the contrasting effects on activation kinetics induced by either 10 or 20 µM NS1643 should contribute to the contrasting effects on the amplitude of the initial current peak produced by these different NS1643 concentrations (see also Fig. 1Bb).

### Effect of NS1643 on Deactivation using Fully Activated Kv11.3 Channels

**Figure 3:**
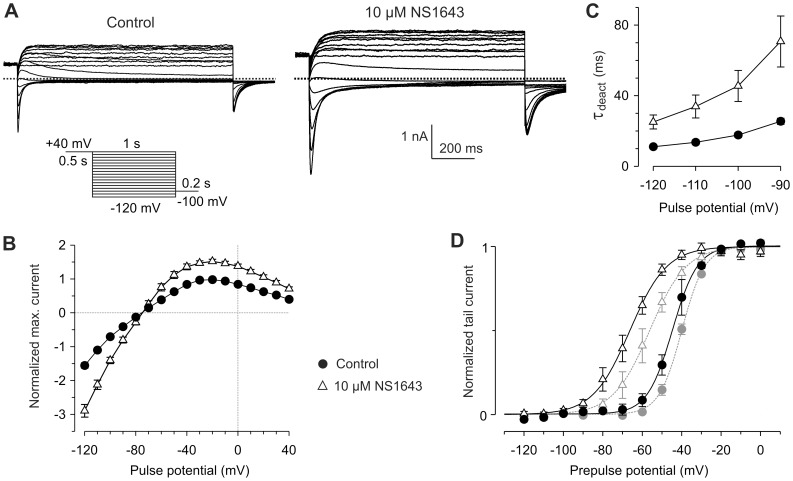
NS1643 enhances fully activated Kv11.3 currents and slows deactivation. From a holding potential of −20 mV, membrane currents were elicited by 0.5 s depolarizations to +40 mV followed by variable 1 s test pulses from +40 to −120 mV with 10 mV decrements and a final step to −100 mV (see pulse protocol). (A) Membrane currents recorded from the same cell under control conditions and in the presence of 10 µM NS1643. (B) To obtain the fully-activated Kv11.3 current-voltage relationship, maximal current amplitudes during the test pulses were plotted as a function of the test pulse potential. Current amplitudes recorded before and after application of 10 µM NS1643 were normalized to the maximal amplitude of the control outward currents. Data points are means ± SEM of paired experiments *(n* = *9*). (C) Time constants of fast deactivation (τ_deact_) as a function of the test pulse potential before and after application of 10 µM NS1643; *n* = 7; *p*<0.05 for all potentials. (D) Normalized tail current amplitudes as a function of the preceding test pulse potential before and after application of 10 µM NS1643. Data were fitted with Boltzmann functions to yield the availability curves. Means ± SEM of paired experiments (*n* = 4). Activation curves obtained from the same cells are included in the diagram in gray.

To analyze the activating effect of 10 µM NS1643 in more detail, experiments were performed with an availability protocol (Fig. 3). Starting from a holding potential of −80 mV, depolarizing pulses to +40 mV were applied to fully activate the Kv11.3 channels, followed by variable 1 s test pulses between +40 and −120 mV with 10 mV decrements. Subsequent steps to −100 mV were applied, eliciting tail currents. Since the majority of Kv11.3 channels were inactivated during the depolarizing prepulse, the variable test pulses induced voltage-dependent recovery from inactivation and deactivation.

Fig. 3A, B shows that 10 µM NS1643 considerably increased the fully activated Kv11.3 currents at all test potentials. The peak inward currents were even more enhanced than the outward currents which could result from a slowing of Kv11.3 channel deactivation kinetics. In fact, 10 µM NS1643 induced a significant slowing of the time course of deactivation. In the analyzed potential range between −90 and −120 mV, τ_deact_ increased to about 250% of the control values (Fig. 3C). As already suggested by the inspection of the tail currents evoked with the envelope-of-tail protocol (see Fig. 2Ab), the higher concentration of 20 µM NS1643 induced an additional pronounced slowing of deactivation. With 20 µM NS1643, τ_deact_ ranged from 111±9 ms at −120 mV to 162±17 ms at −90 mV (*n* = 4; *p*<0.01 for all potentials vs. unpaired data obtained with 10 µM NS1643). A slight increase in the time constants of deactivation already occurred with 1 µM NS1643 (τ_deact_ at −120 mV: 12.5±0.4 ms (control), 17.1±2.2 ms (NS1643) *p* = 0.08; τ_deact_ at −90 mV: 26.5±0.5 ms (control), 42.1±3.2 ms (NS1643) *p* = 0.026; *n* = 3, paired experiments).

The ability of 10 µM NS1643 to enhance the fully activated Kv11.3 currents demonstrates that the drug-induced current increase observed with the activation protocol could not only be due to the left shift in the voltage dependence of channel activation. This left shift was determined using depolarizing test pulses of 4-s duration, and the drug-induced acceleration of Kv11.3 activation kinetics might have contributed to the shift of the activation curves along the voltage axis. True steady-state activation curves are in between isochronal activation and deactivation curves [23]. Therefore, we constructed deactivation (or availability) curves from the tail currents elicited with the availability protocol. In addition, 4-s isochronal activation curves were obtained from the same cells in the absence as well as in the presence of 10 µM NS1643 as shown in Fig. 1. The combined results of these experiments are illustrated in Fig. 3D. The V_0.5_ values for isochronal activation (−39.9±0.8 mV, *n* = 4) and availability curves (−44.5±2.2 mV) only slightly differed under control conditions, suggesting that 4-s depolarizing test pulses are sufficiently long to investigate quasi steady-state activation of Kv11.3 channels. Following treatment with 10 µM NS1643, the distance between the isochronal activation and deactivation curves increased (activation: V_0.5_ = −56.2±3.2 mV; availability: V_0.5_ = −67.3±3.2 mV), which can be readily explained by the drug-induced slowing of deactivation that impaired the reaching of a steady state during the variable 1-s test pulses of the availability protocol.

### Effect of NS1643 on the Inactivation of Kv11.3 Channels

**Figure 4:**
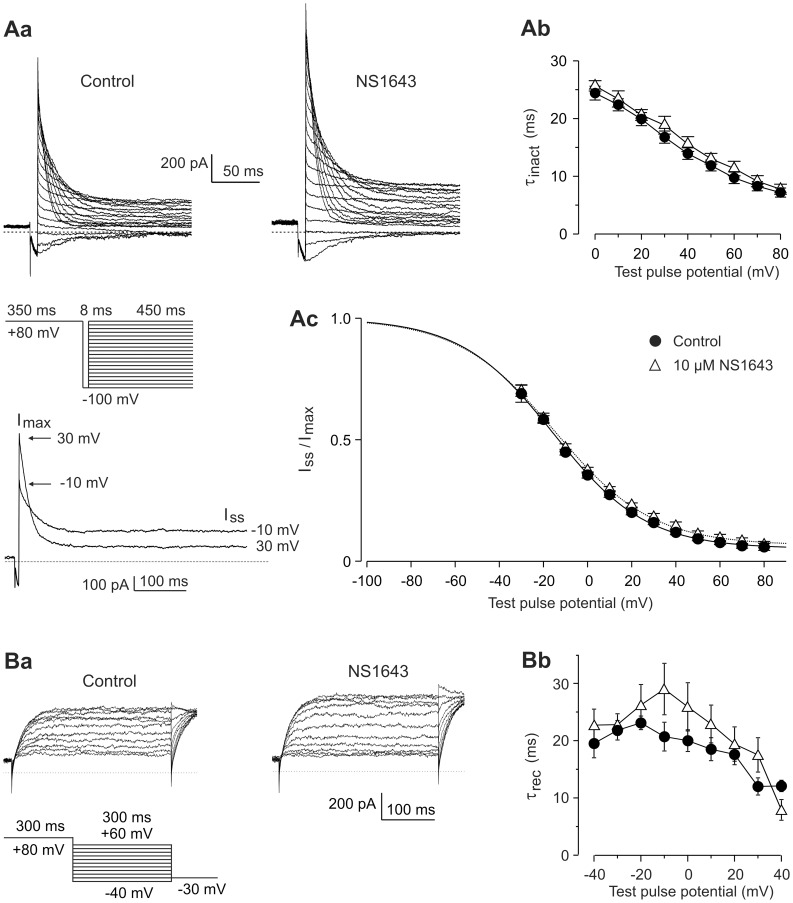
NS1643 has no significant effect on Kv11.3 inactivation and recovery from inactivation. (A) Inactivation of Kv11.3 channels was investigated by a triple pulse protocol. Starting from a holding potential of −80 mV, the potential was stepped to +80 mV (P1) for 350 ms. A subsequent 8 ms P2 pulse to −100 mV was followed by variable 450 ms test pulses to potentials between +80 and −100 mV (P3), with 10 mV decrements (see pulse protocol). (Aa) Current traces obtained before and after application of 10 µM NS1643. (Ab) Time constants of inactivation obtained from monoexponential fits to the decay phase of the current traces. Data are means ± SEM of paired experiments (*n = 5*). (Ac) As a measure of voltage-dependent inactivation, the ratio of the steady-state (I_ss_) to the instantaneous current amplitude (I_max_) elicited with the variable P3 pulse was plotted against the P3 pulse potential and data obtained before and after application of 10 µM NS1643 (*n = 5*) were fitted with a Boltzmann function. I_max_ was measured directly after the capacitance transient (0.4–0.5 ms after the peak) and I_ss_ was determined as mean current level during the last 50 ms of the P3 pulse (see inset beneath the pulse diagram). Current ratios for potentials more negative than −30 mV were not considered due to increasing channel deactivation at these potentials. (B) The time course of recovery from inactivation was investigated with variable repolarizing test pulses starting from the fully activated state of the Kv11.3 channels. (Ba) Current traces recorded with the indicated pulse protocol from the same cell in the absence and in the presence of 10 µM NS1643. (Bb) Means of the time constants of recovery from inactivation (τ_recovery_) as a function of the membrane potential (*n* = 6).

The voltage dependence and time course of inactivation of Kv11.3 channels was analyzed by a triple pulse protocol as shown in Fig. 4A. Starting from −80 mV with a strong depolarizing pulse to +80 mV (P1) leading to full activation and to almost complete inactivation, Kv11.3 channels were recovered from inactivation by a constant 8 ms hyperpolarization to −100 mV (P2). The third pulse (P3) was the final variable test pulse to potentials between +80 and −100 mV, inducing potential-dependent inactivation at the more positive potentials or deactivation at the more negative test pulse potentials. In the potential range between 80 and 0 mV, the current decay during the P3 test pulses was fitted by single exponential functions to achieve the time constants of inactivation (τ_inact_). The time course of current inactivation was not significantly altered by application of 10 µM NS1643 (Fig. 4Ab).

As a measure of voltage-dependent inactivation, we determined the ratio of the steady-state current I_ss_ to the maximal current I_max_ during the P3 pulse (see inset beneath the pulse diagram in Fig. 4A). Boltzmann functions were fitted to the current ratios obtained at P3 potentials without obvious impact of deactivation (−30 to +80 mV), yielding inactivation curves with similar inflection potentials (Control: −15.4±1.1 mV; 10 µM NS1643: −15.1±1.7 mV; *n* = 5, p = 0.91). The slope factors *k* did not significantly differ (Control: −20.8±0.9 mV; 10 µM NS1643: −21.9±1.2 mV; *n* = 5, p = 0.067). In these experiments, the duration of the P2 pulse was a compromise between maximal recovery from inactivation and minimal channel deactivation. Since the duration of P2 was too short to allow complete recovery from inactivation, the obtained inactivation curves do not represent Kv11.3 steady state behavior. Nevertheless, the lack of effect of 10 µM NS1643 on the midpoint of these isochronal inactivation curves suggests that the current increase induced by 10 µM NS1643 did not result from a shift in the voltage dependence of steady-state inactivation to more depolarized potentials.

The effect of 10 µM NS1643 on the kinetics of recovery from inactivation was evaluated in experiments on fully-activated Kv11.3 channels in the voltage range between 60 and 0 mV where the time course of recovery from inactivation was not contaminated by channel deactivation. As shown in Fig. 4B, application of 10 µM NS1643 did not consistently change the time course of recovery from inactivation.

**Figure 5:**
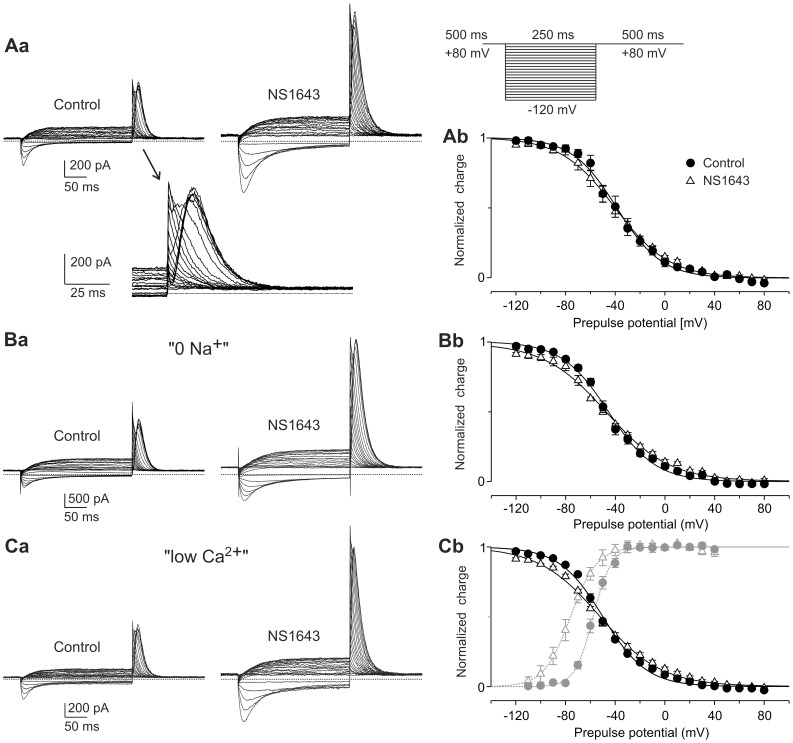
NS1643 increases fully activated Kv11.3 currents without shifting steady state inactivation curves. (A) The voltage dependence of steady-state inactivation of Kv11.3 channels was investigated by a triple pulse protocol consisting of two 500 ms depolarizations to +80 mV (P1 and P3), separated by a variable 250 ms test pulse to potentials between +80 and −120 mV (P2) to induce voltage dependent recovery from inactivation. (Aa) Current traces obtained before and after application of 10 µM NS1643. Control current transients obtained at the beginning of the P3 pulses are also shown on expanded time and voltage scale. (Ab) Means ± SEM (*n = 5*) of the charge flowing during the current transient elicited upon the P3 pulse are plotted against the preceding P2 test pulse potential. Data obtained before and after addition of NS1643 were normalized to the amplitude of the respective Boltzmann functions fitted to the charge values. (B, C) Data from similar experiments performed in external solution without Na^+^ (B, *n = 4*) or with low Ca^2+^ (C, *n = 4*). (Cb) Activation curves obtained from the same cells with an activation protocol as shown in Fig. 1 are included in the diagram in gray.

To further assess the effects of NS1643 on Kv11.3 steady state inactivation, experiments with another triple pulse protocol were performed, consisting of a constant P1 and P3 potential of +80 mV to attain complete channel activation and inactivation and a 250 ms variable P2 test pulse to allow the recovery process to reach a voltage-dependent steady state (Fig. 5). Assuming a linear gating model for Kv11.3 as described for Kv11.1 without direct transitions between the closed and the inactivated state [24], [25], the integral of the transient current (the charge) elicited by the P3 pulse to +80 mV should reflect the relative number of channels that recovered from inactivation during the P2 pulse. Independent of whether these Kv11.3 channels are still open or already deactivated at the end of the variable P2 pulse, they should contribute to the P3 current integral, since deactivated channels will pass again the open state before entering the inactivated state.

Fig. 5A shows that application of 10 µM NS1643 clearly enhanced the fully activated Kv11.3 currents elicited with the P2 pulses as well as the transient P3 currents. The maximum P3 charge measured after the most negative P2 pulses was significantly increased to 184±15% of the respective control values (*n* = 5, *p* = 0.0086; data corrected for slight changes in the inactivation time course). Nevertheless, the potential of half maximal charge - suggested to represent half maximal channel inactivation - did not change (control: −38.7±4.2 mV; NS1643: −41.9±4.1 mV; *p* = 0.34). The decrease in the steepness of the inactivation curve was not significant (control: 16.9±0.3 mV; NS1643∶21.8±2.6 mV; *p* = 0.073).

### Influence of External Na^+^ and Ca^2+^

The results described above indicate that the NS1643-induced increases in macroscopic Kv11.3 conductance in the fully-activated state cannot be explained by reduced inactivation and must involve other mechanisms. For Kv11.1a, the classical HERG channel, external Ca^2+^ as well as external Na^+^ have been found to inhibit the channel and external binding sites for these ions have been discussed [26], [27]. To test whether NS1643 might exert part of its activating effects by a relief of the Kv11 current inhibition produced by these external cations, experiments were performed in external zero Na^+^ or low Ca^2+^ solution.

In the external NMDG (0 Na^+^) solution, 10 µM NS1643 also enhanced the fully activated Kv11.3 currents (Fig. 5B). After drug exposure, the average charge during the P3 pulse amounted to 157±14% (*n* = 4) of the control values, which was not significantly different from the charge increase measured in normal Ringer solution (*p* = 0.23). The potential of half maximal inactivation did not change significantly (control: −45.4±1.9 mV; NS1643: −47.6±1.0 mV; *p* = 0.36). Again, there was an increase in the mean value of the slope factor *k*, which was not significant (control: 18.6±1.3 mV; NS1643∶25.9±2.2 mV; *p* = 0.076).

Also with external free Ca^2+^ buffered to submicromolar values, application of 10 µM NS1643 resulted in an increase in the fully activated Kv11.3 currents (Fig. 5C). Very similar to the corresponding normal Ringer data, the P3 charge increased to 185±18% (*n* = 4, *p* = 0.0086) of the control values. The potential of half maximal inactivation did not change (control: −49.8±1.3 mV; NS1643: −51.9±1.4 mV; *p* = 0.18), but there was a significant increase in the slope factor *k* (control: 17.5±0.5 mV; NS1643∶24.8±1.8 mV; *p* = 0.022), indicating a reduced voltage sensitivity of channel inactivation.

In low Ca^2+^ solution, the potential for half maximal Kv11.3 channel inactivation was about 10 mV more negative than that measured in normal Ringer solution. Activation curves determined in the same cells demonstrate, that the voltage dependence of activation was even more affected by external Ca^2+^ buffering than inactivation: with a mean value of V_0.5_ = −57.6±1.3 mV, the activation curve was shifted by almost 20 mV to more negative potentials. Nevertheless, NS1643 was still able to induce a remarkable left-shift in the voltage dependence of activation to V_0.5_ = −77.2±2.1 mV (*n* = 4, *p* = 0.0057; Fig. 5Cb) which was associated with a decrease in the voltage sensitivity of the activation process (control: *k* = 7.3±0.4 mV; NS1643: *k* = 13.0±2.6 mV; *p* = 0.044).

It should be noted, that with prolonged exposure to 10 µM NS1643 (up to 60 min), the antagonistic effects of NS1643 could increase as observed with higher drug concentrations. A delayed decrease in Kv11.3 current was always associated with a further flattening of the inactivation curve and it could include a shift in the potential of half maximal inactivation to more positive values. In addition, similar experiments performed with freshly thawed CHO cells showed a more pronounced antagonistic effect of 10 µM NS1643 suggesting that channel-independent membrane properties might influence the efficacy of the drug.

## Discussion

The small molecule compound NS1643 is a HERG channel activator, which has been found to affect the widespread splice variants Kv11.1a and Kv11.1b [14], [16], [17], [18], [28] as well as the Kv11.2 channel [20]. We have now investigated the NS1643-induced changes in the biophysical properties of rat Kv11.3 channels using CHO cells as a mammalian expression system. We found that Kv11.3 current amplitudes increased in a voltage- and dose-dependent manner up to about twofold for the maximal steady-state current at a concentration of 10 µM NS1643. The most obvious changes in the biophysical properties suited to contribute to the current increase were a leftward shift in the voltage dependence of activation, an acceleration of activation as well as a slowing of deactivation. Concentrations higher than 10 µM revealed antagonistic drug effects which were accompanied by a pronounced decrease in the voltage sensitivity of Kv11.3 channel activation with concomitant slowing of activation and deactivation kinetics.

### Voltage Dependence of Activation and Gating Kinetics

In the concentration range between 0.1 and 10 µM, NS1643 induced dose-dependent left shifts in the Kv11.3 activation curve. These changes imply a more negative activation threshold and more steady-state current in the negative potential range. The inflection potentials of isochronal activation curves of Kv11 channels depend on both, the duration of the depolarizing test pulses as well as on the activation kinetics. Accelerated activation results in a left shift of the isochronal activation curve if the test pulse duration is too short. In our experiments, only small differences were found between isochronal activation and deactivation curves, due to the relatively faster activation and deactivation kinetics of Kv11.3 compared to Kv11.1 channels [1], [2], [29], [30]. Thus, the shifts in the 4s-isochronal activation curves induced by NS1643 concentrations of up to 10 µM should represent similar changes in the true steady-state I-V relationship of Kv11.3 channel activation. The most prominent effects of NS1643 on Kv11.3 gating kinetics were related to the activation and the deactivation process, whereas no significant alterations were found for the time course of inactivation and recovery from inactivation. Acceleration of activation with essentially unchanged inactivation kinetics should contribute to the prominent increase in the Kv11.3 current transient induced by 10 µM NS1643. Kv11.3 deactivation kinetics were slowed in a dose-dependent manner in the concentration range from 1 to 20 µM NS1643. The slowing of both, activation and deactivation kinetics by 20 µM NS1643 is consistent with the strong decrease in the voltage sensitivity of the channel produced by this high drug concentration.

### Effects on Kv11.3 Channel Inactivation

A thorough investigation was conducted on the effects of NS1643 on the voltage dependence of Kv11.3 inactivation, because the HERG activator has first been described to mainly produce its Kv11.1a current activating effects by shifting the steady-state inactivation to more positive potentials, resulting in reduced channel inactivation [14], [16], [19].

Different methods have been used for Kv11.1 channels to measure the voltage dependence of steady-state inactivation, resulting in significantly differing V_0.5_ values (reviewed in [9]). Most of these methods are less suited to investigate Kv11.3 channel inactivation due to its faster deactivation kinetics limiting the maximal fraction of open channels achieved upon re- or hyperpolarizing pulses. In the present study, the effect of NS1643 on Kv11.3 channel inactivation was assessed using two different triple pulse protocols. The first protocol (illustrated in Fig. 4A) has often been used to directly demonstrate the voltage dependence of HERG channel inactivation and to analyze inactivation kinetics [24], [31]. With this protocol, the ratio of the P3 steady-state current to the P3 instantaneous current amplitude is a measure of voltage-dependent inactivation during the variable P3 pulse at potentials which do not induce channel deactivation [2], [30]. The resulting values do not represent the “true” steady-state inactivation of Kv11.3, because the duration of the P2 pulse was too short to induce complete recovery from inactivation. This resulted in submaximal instantaneous current amplitudes and thus in higher values for the ratio of the sustained current to the peak current elicited with the P3 pulse. On the other hand, the P2 pulse could not be prolonged to avoid substantial channel deactivation - especially since drug application would diminish the proportion of channels deactivated during P2. Thus, the potential for half maximal steady-state inactivation must be somewhat more negative than the inflection potential of the isochronal inactivation curves derived from this protocol. Nevertheless, as NS1643 did not alter the recovery kinetics, the insufficient time for complete recovery should have equally affected the Kv11.3 currents under control conditions and in the presence of NS1643. Since the resulting isochronal inactivation curves were very similar, it seems unlikely that NS1643 remarkably changed the voltage dependence of Kv11.3 steady-state inactivation.

The second pulse protocol used to study inactivation was a modified availability protocol (see Fig. 5). This new triple pulse inactivation protocol was especially designed for Kv11.3, which exhibits nicely measurable transient currents upon strong depolarizing pulses due to its faster activation and slightly slower inactivation kinetics compared to Kv11.1 [1], [2], [29], [30], [32]. A characteristic of all three members of the Kv11 channel family is their peculiar gating behaviour conferring inward-rectifying properties on these voltage-dependent channels (reviewed in [3], [33]). The kinetics of inactivation and recovery from inactivation are quite fast compared to activation and deactivation kinetics and the channels have to pass the open state to inactivate upon depolarization and to close upon repolarization. The applicability of the linear model to describe the macroscopic currents of Kv11.1 channels [24] has only recently been confirmed [25]. Assuming that the linear model also applies to Kv11.3 channels, the charge-potential curves recorded in the present study should closely represent Kv11.3 steady-state inactivation.

These quasi steady-state inactivation curves were not shifted along the voltage axis, but a reduction in the voltage sensitivity indicated by significantly increased values of the slope factor *k* was found in part of the experiments. A weaker voltage sensitivity of the inactivation process without any shift in the midpoint of the inactivation curve implies relatively more inactivation at more negative potentials and less inactivation at more positive potentials. Even a slight decrease in inactivation at voltages more positive than about −40 mV would favour increased current amplitudes of fully activated Kv11.3 currents. Thus, a drug-induced reduction in the voltage sensitivity of inactivation might contribute to the observed Kv11.3 current activating properties of NS1643.

The drug-induced decrease in the voltage sensitivity of inactivation fits well with the reduced voltage sensitivity of channel activation already produced by 10 µM NS1643. Nevertheless, with or without drug, the steepness of activation and inactivation curves strongly differ for Kv11.3, with the slope factor considerably smaller (voltage sensitivity greater) for activation than inactivation. This is consistent with the existence of distinct voltage sensors for these two processes as proposed for Kv11.1 [34]. Independent voltage sensors can also explain the ability of NS1643 to shift the voltage dependence of Kv11.3 activation without changing the potential for half maximal inactivation.

### Comparison with the Action of NS1643 on Other Members of the Kv11 Channel Family

Most studies on the mechanism of Kv11 channel activation by NS1643 have been performed in *Xenopus* oocytes (e.g. [14], [20], [28]). Since NS1643 has a higher potency and exerts some qualitatively different actions when mammalian cells were used as expression system [18], we mainly compare the present results with data obtained in mammalian cells.

NS1643 affected Kv11.3 channels essentially in the same concentration range as determined for Kv11.1, since 0.1 µM NS1643 already induced significant left shifts in Kv11 activation curves and maximum current increases occurred with about 10 µM NS1643 [17], [18]. In addition, the molecular mechanisms underlying Kv11.3 and Kv11.1 current increases are similar with NS1643 affecting channel activation rather than inactivation [18]. Almost the same mechanism of action has been described for the HERG channel activator mallotoxin which was found to induce a leftward shift in the Kv11.1a activation curve combined with accelerated activation and decelerated deactivation kinetics. In addition, it produces a reduction in the slope of the inactivation curve without any voltage shift [35].

Differences in the NS1643 effects on Kv11.3 and Kv11.1 channels concern the magnitude of the agonistic effects and the action of higher drug concentrations. For example, the near optimum concentration of 10 µM NS1643 induced a current increase to about twofold for Kv11.3 compared to about fourfold for Kv11.1b. Also the leftward shift in the voltage dependence of activation is less pronounced for Kv11.3 than for Kv11.1 channels [18]. A partial antagonistic action of NS1643 has first been described for Kv11.1 channels expressed in oocytes where the drug-induced current increase was attenuated by 100 µM NS1643 [14]. Consistent with a higher potency of NS1643 in mammalian cells, an adverse effect on the drug-induced increase in Kv11.1b current amplitude was already found with 30 µM NS1643 [18] which is comparable to the present data on Kv11.3 channels. However, the partial antagonistic effects of 20 µM NS1643 on Kv11.3 channels were especially pronounced, resulting even in current amplitudes below the control values for the Kv11.3-characteristic transient current component. At this concentration, the leftward shift in the potential of half maximal channel activation completely reversed and the voltage sensitivity of Kv11.3 channel activation drastically decreased. In contrast, NS1643-induced leftward shifts in Kv11.1 activation curves do not saturate or reverse [18] and they are not associated with changes in the voltage sensitivity of channel activation. Thus, the pronounced loss of voltage sensitivity found in the present study can be regarded as a special characteristic of the action of NS1643 on the Kv11.3 channel.

NS1643 has also been found to activate Kv11.2 channels [20]. Qualitatively similar to the present results, NS1643 affects Kv11.2 channel activation, but deactivation is not slowed. Although obtained in different expression systems, these data suggest that NS1643 activates Kv11.2 and Kv11.3 channels by slightly different mechanisms. The action of another small molecule HERG activator, RPR26024, has been investigated on Kv11.3 channels. Interestingly, this drug lacked an agonistic activity on Kv11.3 and even blocked Kv11.3 channels when higher concentrations were used [21], [22]. Also niflumic acid has been found to activate the three Kv11 channels to a differing degree [36]. These channel subtype-specific differences in drug effects advise to investigate the specific actions of HERG activators on all three Kv11 channels.

### Mechanism of Action

The present experiments suggest the existence of more than one mechanism of NS1643 to affect the biophysical properties of Kv11.3 channels. For Kv11.1, different actions of NS1643 seem to involve different binding sites [14], [19], [37]. Interestingly, a single amino acid replacement in the outer mouth of the selectivity filter, which is highly conserved within the Kv11 family, has recently been found to abolish the NS1643-induced shift in the activation of Kv11.1 to more hyperpolarized potentials [38]. In the absence of a rightward shift in the voltage dependence of inactivation, the NS1643-induced increases in the amplitude of fully-activated Kv11.3 currents cannot be completely explained by a possible small decrease in the voltage sensitivity of channel inactivation leading to reduced inactivation at more positive potentials. Thus, increases in single channel conductance or maximal open probability might contribute to the increase in macroscopic Kv11.3 current.

It has been shown that rises in external K^+^ enhance the conductance of all three Kv11 channels [2]. The mechanism of the unusual K^+^ dependence of Kv11.1 has been explained by a channel blocking effect of external Na^+^ ions which is relieved by rises in external K^+^ [27], [39]. We have now found that NS1643 is able to increase fully-activated Kv11.3 currents in the absence of external Na^+^, suggesting that the drug-induced increase in macroscopic Kv11.3 channel conductance is not mediated by a decrease in the binding affinity of Na^+^ ions. The Kv11.1 channel also exhibits an unusually high sensitivity to external divalent cations, and a reduction in the external free Ca^2+^ concentration increases Kv11.1 current by mechanisms strikingly similar to those induced by 10 µM NS1643 in the present study [26], [34], [40], [41]. We found that buffering the external free Ca^2+^ concentration to a submicromolar level shifted the voltage dependence of Kv11.3 channel activation by about 20 mV to more negative potentials. The inactivation curve shifted leftward by only about 10 mV, suggesting that a reduction in external Ca^2+^ results in an increased amplitude of Kv11.3 steady-state current with a maximum at more negative potentials. Despite these pronounced effects of Ca^2+^ buffering, NS1643 was still able to induce increases in the fully-activated Kv11.3 currents and a further leftward shift in the voltage dependence of activation. This demonstrates, that the Kv11.3 channel activating effects of NS1643 are not mediated by a relief of the channel inhibiting properties of Ca^2+^.

### Physiological Context

The present results show that NS1643 exerts distinct agonistic and partial antagonistic effects on the three members of the Kv11 family. Thus, the effect of this drug on the electrical properties of Kv11 channel expressing neurones in the central nervous system might differ considerably depending on the relative expression level of the different Kv11 family members and the achieved drug concentration. On the one hand, a contribution of Kv11.3 channels to the resting membrane potential should always be enhanced by NS1643, because it shifted the activation threshold to more negative values even at high drug concentration. Thus, NS1643 probably stabilizes the resting potential leading to reduced cellular excitability. On the other hand, the contribution of Kv11.3 channels to the phenomenon of action potential frequency accommodation [12] would be differently affected by lower and higher NS1643 concentrations since the Kv11.3 peak current is essential for a fast onset of spike frequency adaptation. Thus, 10 µM NS1643 should enhance frequency adaptation, whereas 20 µM NS1643 might even support sustained firing.

The physiological role of the different Kv11 channels in the central nervous system is still a matter of investigation and at present, it is completely unclear whether Kv11 channels are suited as pharmacological target to influence neuronal excitability outside the lab. Nevertheless, drugs specifically affecting a given Kv11 subunit would probably diminish its unwanted side effects either in the heart or in the brain.
